# Burning plasma achieved in inertial fusion

**DOI:** 10.1038/s41586-021-04281-w

**Published:** 2022-01-26

**Authors:** A. B. Zylstra, O. A. Hurricane, D. A. Callahan, A. L. Kritcher, J. E. Ralph, H. F. Robey, J. S. Ross, C. V. Young, K. L. Baker, D. T. Casey, T. Döppner, L. Divol, M. Hohenberger, S. Le Pape, A. Pak, P. K. Patel, R. Tommasini, S. J. Ali, P. A. Amendt, L. J. Atherton, B. Bachmann, D. Bailey, L. R. Benedetti, L. Berzak Hopkins, R. Betti, S. D. Bhandarkar, J. Biener, R. M. Bionta, N. W. Birge, E. J. Bond, D. K. Bradley, T. Braun, T. M. Briggs, M. W. Bruhn, P. M. Celliers, B. Chang, T. Chapman, H. Chen, C. Choate, A. R. Christopherson, D. S. Clark, J. W. Crippen, E. L. Dewald, T. R. Dittrich, M. J. Edwards, W. A. Farmer, J. E. Field, D. Fittinghoff, J. Frenje, J. Gaffney, M. Gatu Johnson, S. H. Glenzer, G. P. Grim, S. Haan, K. D. Hahn, G. N. Hall, B. A. Hammel, J. Harte, E. Hartouni, J. E. Heebner, V. J. Hernandez, H. Herrmann, M. C. Herrmann, D. E. Hinkel, D. D. Ho, J. P. Holder, W. W. Hsing, H. Huang, K. D. Humbird, N. Izumi, L. C. Jarrott, J. Jeet, O. Jones, G. D. Kerbel, S. M. Kerr, S. F. Khan, J. Kilkenny, Y. Kim, H. Geppert Kleinrath, V. Geppert Kleinrath, C. Kong, J. M. Koning, J. J. Kroll, M. K. G. Kruse, B. Kustowski, O. L. Landen, S. Langer, D. Larson, N. C. Lemos, J. D. Lindl, T. Ma, M. J. MacDonald, B. J. MacGowan, A. J. Mackinnon, S. A. MacLaren, A. G. MacPhee, M. M. Marinak, D. A. Mariscal, E. V. Marley, L. Masse, K. Meaney, N. B. Meezan, P. A. Michel, M. Millot, J. L. Milovich, J. D. Moody, A. S. Moore, J. W. Morton, T. Murphy, K. Newman, J.-M. G. Di Nicola, A. Nikroo, R. Nora, M. V. Patel, L. J. Pelz, J. L. Peterson, Y. Ping, B. B. Pollock, M. Ratledge, N. G. Rice, H. Rinderknecht, M. Rosen, M. S. Rubery, J. D. Salmonson, J. Sater, S. Schiaffino, D. J. Schlossberg, M. B. Schneider, C. R. Schroeder, H. A. Scott, S. M. Sepke, K. Sequoia, M. W. Sherlock, S. Shin, V. A. Smalyuk, B. K. Spears, P. T. Springer, M. Stadermann, S. Stoupin, D. J. Strozzi, L. J. Suter, C. A. Thomas, R. P. J. Town, E. R. Tubman, C. Trosseille, P. L. Volegov, C. R. Weber, K. Widmann, C. Wild, C. H. Wilde, B. M. Van Wonterghem, D. T. Woods, B. N. Woodworth, M. Yamaguchi, S. T. Yang, G. B. Zimmerman

**Affiliations:** 1 Lawrence Livermore National Laboratory, Livermore, CA USA; 2 Los Alamos National Laboratory, Los Alamos, NM USA; 3 Laboratoire pour l’utilisation des Lasers Intenses chez École Polytechnique, Palaiseau, France; 4 Laboratory for Laser Energetics, University of Rochester, Rochester, NY USA; 5 General Atomics, San Diego, CA USA; 6 Massachusetts Institute of Technology, Cambridge, MA USA; 7 SLAC National Accelerator Laboratory, Menlo Park, CA USA; 8 Atomic Weapons Establishment, Aldermaston, UK; 9 Diamond Materials, Freiburg, Germany

**Keywords:** Nuclear fusion and fission, Laser-produced plasmas

## Abstract

Obtaining a burning plasma is a critical step towards self-sustaining fusion energy^1^. A burning plasma is one in which the fusion reactions themselves are the primary source of heating in the plasma, which is necessary to sustain and propagate the burn, enabling high energy gain. After decades of fusion research, here we achieve a burning-plasma state in the laboratory. These experiments were conducted at the US National Ignition Facility, a laser facility delivering up to 1.9 megajoules of energy in pulses with peak powers up to 500 terawatts. We use the lasers to generate X-rays in a radiation cavity to indirectly drive a fuel-containing capsule via the X-ray ablation pressure, which results in the implosion process compressing and heating the fuel via mechanical work. The burning-plasma state was created using a strategy to increase the spatial scale of the capsule^2,3^ through two different implosion concepts^4–7^. These experiments show fusion self-heating in excess of the mechanical work injected into the implosions, satisfying several burning-plasma metrics^3,8^. Additionally, we describe a subset of experiments that appear to have crossed the static self-heating boundary, where fusion heating surpasses the energy losses from radiation and conduction. These results provide an opportunity to study α-particle-dominated plasmas and burning-plasma physics in the laboratory.

## Abstract (web summary)

A burning plasma, a critical step towards self-sustaining fusion, is achieved at the US National Ignition Facility, with a subset of experiments demonstrating fusion self-heating beyond radiation and conduction losses.

## Main

Fusion research fundamentally aims to create a system that produces more energy than was required to create it, a necessary condition for energy applications; in practice, the fusion reaction must be self-sustaining, with self-heating overtaking loss mechanisms, termed ‘ignited’^9^. Such conditions are reached in astrophysical objects including the cores of stars, novae and type 1a supernovae, and in thermonuclear weapons. Ignition in the laboratory requires heating the fuel to incredibly high temperatures, where it becomes a ‘plasma’ and fusion reactions readily occur, while also controlling energy losses. Several approaches have been developed to heat and confine plasma over the past several decades, with most pursuing deuterium–tritium (DT) fuel, which most easily achieves ignition. The dominant approaches to plasma confinement are ‘inertial’, an impulsive burn while the fuel is confined by its own inertia, and ‘magnetic’, in which specialized configurations of magnetic fields provide confinement to the charged particles in the plasma. In order for a DT fusion (D + T → *α* (3.5 MeV) + *n* (14 MeV)) plasma to become thermally unstable and ignite, it must first obtain a ‘burning’ state. In this regime, self-heating from α-particle deposition exceeds the external heating input into the DT^8^; this ratio is denoted *Q*_α_, where the self-heating is taken relative to the heating power to the plasma—for inertial fusion this is the *P*d*V* compressional work on the fuel and not the total laser energy (*P*, pressure, d*V*, volume change). *Q*_α_ > 1 is a burning plasma.

A burning-plasma state signifies a transformational change to the energy and power balance in the DT plasma, opening up the potential for rapidly increasing performance. In the impulsive case of inertial confinement fusion (ICF)^10^, *Q*_α_ can be stated either as a power during burn, or as an energy integrated over the burn duration, whereas for the near-steady-state operation of magnetic fusion energy (MFE), *Q*_α_ is a statement of power. As α-particles carry 1/5 of the total fusion energy per D + T reaction, *Q*_α_ = *Q*/5, where *Q* is the total fusion energy compared to the heating energy supplied. (Or in the MFE case, stated in terms of total fusion power over heating power; for example, the goal of ITER^11^ is to reach *Q*_α_ ≈ 2 (*Q* ≈ 10), whereas the record from the JET tokamak^12^ is *Q*_α_ ≈ 0.13 (*Q* ≈ 0.67).)

A burning plasma is distinct from other scientific milestones in inertial fusion. In 2014, the first milestone of ‘fuel gain’^13^ (*G*_fuel_ > 1) was achieved^14^, in which the fusion yield exceeds the energy delivered to the fuel; this corresponds to approximately 12–14 kJ of yield at the National Ignition Facility (NIF). At 20–22 kJ, the yield was approximately doubled by self-heating feedback, termed ‘α-heating’^15^. The next scientific milestone is a burning plasma, as described previously; this is the scientific milestone achieved in this work. No net energy gain, *G*, relative to the laser energy is expected for a burning plasma. Because of energy losses incurred in achieving the required compressed state, ICF implosions must achieve ignition before a net energy gain is possible. A net energy gain would require fusion yields greater than the laser energy, 1.9 MJ. Although short of ignition or energy gain, a burning plasma (*Q*_α_ > 1) is a new physics regime for laboratory fusion^1,11,16^. Studying burning plasmas will elucidate other new physics in this regime, such as self-heating-driven instabilities or kinetic effects in the plasma, which probably depend on the confinement approach.

**Fig. 1:**
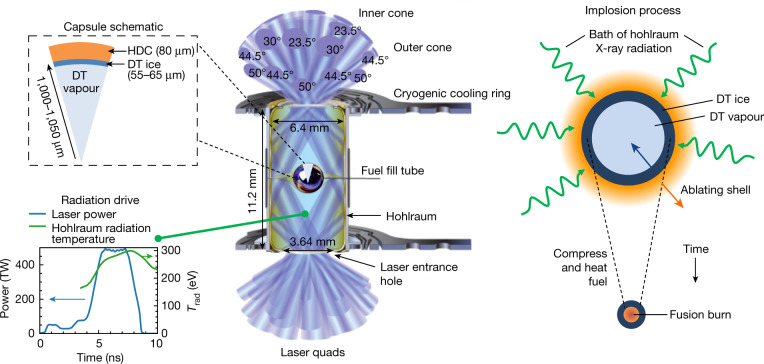
Schematic of the indirect-drive inertial confinement approach to fusion. Centre, A typical indirect-drive target configuration with key engineering elements labelled. Laser beams (blue) enter the hohlraum through laser entrance holes at various angles. Top left, A schematic pie diagram showing the radial distribution and dimensions of materials in diamond (high-density carbon, HDC) ablator implosions. Bottom left, The temporal laser power pulse-shape (blue) and associated hohlraum radiation temperature (green). Right, At the centre of the hohlraum, the capsule is bathed in X-rays, which ablate the outer surface of the capsule. The pressure generated drives the capsule inward upon itself (an implosion) which compresses and heats the fusion fuel during the implosion process.

In a tokamak, the predominant approach to magnetic confinement, once the plasma discharge is generated by resistive heating, external power sources, such as radio-frequency antenna, provide additional plasma heating as the plasma is brought to fusion conditions. In indirect-drive ICF, the way energy is delivered to the fusion fuel is different and much less direct. At NIF^17^, 192 lasers deliver up to 1.9 MJ of frequency-tripled light into a high atomic number (*Z*) ‘hohlraum’ (Fig. 1) that serves the purpose of an X-ray converter generating a nearly Planckian X-ray bath, an approach known as ‘indirect drive’^18^. The incident beam-by-beam laser pointing and power in time are designed^6^ to generate a specific radiation temperature (*T*_rad_) history (Fig. 1, bottom left) inside the hohlraum, with sufficient uniformity in a way that is matched to specifics of the target geometry and the desired final plasma state. The exposed surface of a capsule at the centre of the hohlraum absorbs approximately 10–15% of the X-rays, causing the outer edge of the capsule (the ablator) to ionize, generate high pressures of the order of hundreds of Mbar (1 Mbar = 10^11^ Pa), and expand away from the capsule—a process termed ablation. A shell of cryogenic DT fuel is layered against the inside surface of the ablator, which is in partial-pressure equilibrium with DT vapour in the centre of the capsule (Fig. 1, top left). The inwardly directed acceleration caused by the ablation drives the capsule and DT fuel inwards upon itself (an implosion, shown schematically at the right of Fig. 1) with enormous acceleration (about 10^14^ m s^−2^) obtaining velocities of approximately 350–400 km s^−1^ in a matter of nanoseconds. Most of the X-ray energy (about 92–95%) absorbed by the capsule is consumed by the ablation process, but as a result the DT fuel obtains considerable (about 10–20 kJ) kinetic energy inside a very small volume.

Shortly after the DT fuel acquires peak kinetic energy, the pressure (*P*) inside the implosion rises markedly, to levels of many hundreds of Gbar (1 Gbar = 10^14^ Pa), as kinetic energy is converted into internal energy in the DT (a process termed stagnation). An ICF implosion is a pressure amplifier, sacrificing absorbed energy to achieve high energy density and central pressures that are factors of thousands higher than the pressure at the ablation front. The high central pressure is necessary because only a small fraction of the energy at NIF can ever be coupled into the DT fuel, and heating a large mass of DT fuel is energetically costly, as reflected in the heat capacity of DT, *c*_DT_ = 115 kJ mg^−1^ keV^−1^ (9.9 × 10^3^ J kg^−1^ K^−1^). In these experiments the total fuel mass is approximately 200 μg and the hot-spot mass is approximately 20–30 μg. As a high ion temperature (*T*_i_) is also needed for fusion, while the fuel stagnates at the centre of the implosion, the DT forms a hot spot from the fuel’s inner surface and *P*d*V* work is done on the hot spot, generating very high ion and electron temperatures in near thermal equilibrium (*T*_i_ ≈ *T*_e_ ≈ 4–5 keV, 1 keV = 1.16 × 10^7^ K, where *T*_i_ and *T*_e_ are the ion and electron temperatures). If the conditions of high temperature and pressure are achieved, the hot spot initiates copious DT fusion reactions and self-heating further increases *T*_i_.

ICF experiments have already demonstrated considerable fusion performance enhancement from self-heating^14,15^, and more recent advances^19–21^ have generated experiments with approximately 50 kJ fusion yields that were close to the burning-plasma threshold^3^. These experiments used capsules with similar inner radii, between 0.91 and 0.95 mm. Within the maximum laser energy NIF can deliver, these previous designs were limited in the energy coupled to the capsule, and thus in the fuel kinetic energy, by the ability to control the symmetry of the radiation environment within the hohlraum, primarily because an ablated plasma bubble expands from where the outer beams hit the wall (Fig. 1), intercepting the inner beams and thereby suppressing drive at the hohlraum waist^22,23^. Two tactics have been used to enable symmetry control with more efficient hohlraums driving larger capsules: adjusting cross-beam energy transfer between the outer to inner beams^4,24,25^ by changing the laser wavelength separation (Δ*λ*); and incorporating a pocket in the hohlraum wall at the outer beam location to delay the bubble propagation^5^. These tactics have been used to design higher-efficiency hohlraums that control symmetry; we use these hohlraums to drive capsules that are about 10% larger than prior experiments to realize the strategy for achieving a burning plasma laid out in a previous work^2^. These experimental campaigns are known as ‘Hybrid E’ and ‘I-Raum’; the Hybrid-E campaign uses Δ*λ* exclusively, whereas I-Raum uses a combination of the pocket and Δ*λ*. Key data and analysis supporting this burning-plasma analysis are given in complementary papers: Ross et al.^7^ (experiments) and Kritcher et al.^6^.

Four experiments with these new designs have been conducted that have generated record performance at NIF, with triple the fusion yield compared to past experiments^4,19,21^, to a maximum of approximately 170 kJ reported here. The experiments are referred to by an experiment number denoting the date of the experiment (for example, in the format NYYMMDD, where YY = year, MM = month and DD = day). N201101 and N210207 were experiments using the Hybrid-E platform, and N201122 and N210220 were experiments using the I-Raum platform. The experiments in November (N201101 and N201122) achieved much higher performance relative to past work owing to their increased scale and favourable implosion design parameters, yet each suffered from low-mode degradations; these low-mode asymmetries were mitigated on the subsequent experiments (N210207 and N210220), resulting in higher performance^6,7^.

On each NIF experiment a comprehensive suite of optical, X-ray and nuclear diagnostics measure key aspects of the implosion performance. Key data are shown in Extended Data Table 1: the total fusion yield in kJ, ion temperature (*T*_i_, measured from DD reactions)^7^, hot-spot volume and burn width in ps. For a full description of the experimental data and changes between the experiments, see ref. ^7^. Analytic models using these data are used to infer characteristics of the implosion process and hot spot including the pressure, hot-spot internal energy, implosion velocity and peak kinetic energy in the fuel during implosion, *P*d*V* work done on the hot spot, and areal density of the hot spot in g cm^−2^. These quantities are required to evaluate the burning-plasma criteria. Most of these inferences are described in a previous work^26^ and Methods; the implosion velocity (*v*_imp_) can be inferred from the time of maximum neutron output (‘bang time’) and an implosion dynamics ‘rocket model’ that is calibrated to near-neighbour surrogate experiments in which the implosion trajectory is tracked radiographically^27,28^.

**Fig. 2:**
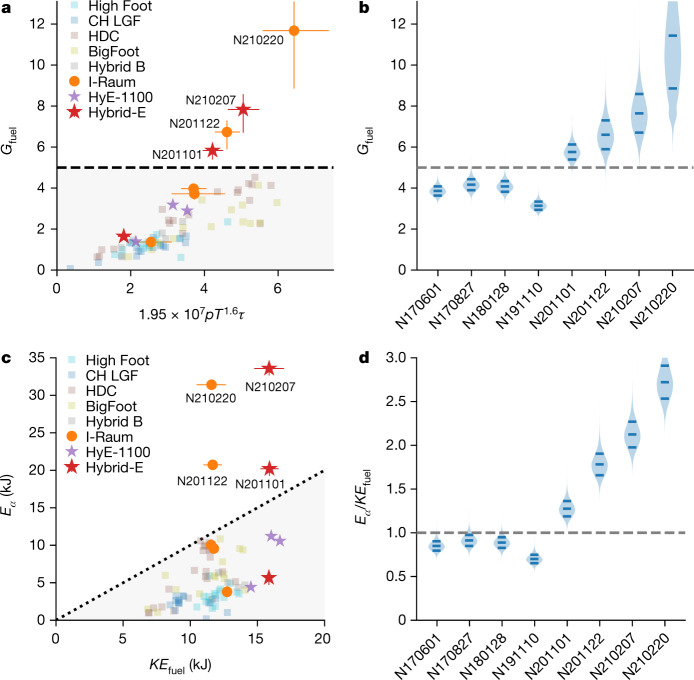
Simple metrics for assessing a burning plasma. **a**, Total fuel gain versus Lawson-like parameter; *G*_fuel_ > 5 corresponds to the burning-plasma regime. **b**, Probability distributions for *G*_fuel_ for high-performing experiments. In these plots the width of the shaded region is proportional to the probability distribution and the solid lines mark the 16th, 50th and 84th percentiles of the distribution **c**, Total α-heating energy versus fuel kinetic energy, *E*_α_/KE_fuel_ > 1 corresponds to *Q*_α_ > 1. **d**, Probability distributions in *E*_α_/KE_fuel_ criteria for high-performing experiments. Error bars in **a**, **c** are 1 standard deviation (s.d.) and are shown only for the I-Raum and Hybrid-E points. Historical data are from refs. ^4,14,15,19–21,29–31^.

Although it would be desirable to have a direct measure that indicates a burning plasma, such a measurement is not yet known to exist, so inferences from data must be used instead. *G*_fuel_ has a direct connection to ignited fusion requirements and suggests a simple metric for assessing a burning plasma from *G*_fuel_ = *Y*/*E*_*P*d*V*,tot_, where *Y* is the fusion yield and *E*_*P*d*V*,tot_ is the total *P*d*V* work on the fuel (see Methods for how this quantity is evaluated). Figure 2a (also Extended Data Table 1) shows a plot of *G*_fuel_ data from many DT implosions at NIF versus the product *PT*^1.6^*τ*, where *τ* is a confinement time; this is a Lawson-like criterion applicable for *G*_fuel_ (Methods).

As can be seen in Fig. 2a, most experimental series show a linear trend between *G*_fuel_ and $P{T}_{{\rm{i}}}^{1.6}\tau$ and have *G*_fuel_ ≤ 5; however, the Hybrid-E and I-Raum implosion series show a transition to a super-linear trend between *G*_fuel_ and $P{T}_{{\rm{i}}}^{1.6}\tau$ (as expected when self-heating exceeds the hot-spot internal energy) and have *G*_fuel_ > 5. The non-burning-plasma regime is denoted by the grey shaded region (*G*_fuel_ < 5). In this and the following figures, historical data from NIF are shown from refs. ^4,14,15,19–21,29–31^, labelled by the names of those predecessor campaigns.

Figure 2b shows the probable distribution of the *G*_fuel_ values plotted in Fig. 2a, with the probability distribution in the inferred data quantities included to evaluate the uncertainty (Methods). For comparison, we include a set of previous high-performing NIF experiments from refs. ^4,19,21^. The abscissa of Fig. 2b are NIF experiment numbers; although several experiments in years prior to November 2020 came very close to the threshold of *G*_fuel_ = 5, only the experiments reported here have so far clearly surpassed it (see Extended Data Table 1 for values, where the quoted likelihood is the fraction of the distribution above the threshold).

Alternatively, comparing the total energy produced in α-particles, *E*_α_ = *Y*/5, to the peak kinetic energy of the DT fuel, KE_fuel_ (Fig. 2c), is another simple metric. Similar to Fig. 2b, Fig. 2d shows the probable range of *E*_α_/KE_fuel_, with normally distributed uncertainties in the input data versus experiment number for the eight highest performing DT experiments at NIF, where again only these four experiments clearly exceed *E*_α_/KE_fuel_ > 1 (see Extended Data Table 1 for values). Because indirect-drive implosions have a small fraction of ablator mass remaining at peak velocity it is important to point out that KE_fuel_ is not the total implosion kinetic energy at peak velocity; instead, because the stagnating shell is compressible and extended radially, only a fraction of the kinetic energy of the ablator can be converted to internal energy before peak burn and disassembly.

Although *G*_fuel_ and *E*_α_/KE_fuel_ are suggestive metrics for an ICF burning plasma, two more rigorous and more stringent metrics already exist in the literature^3,8^. The burning-plasma statement that ‘α-deposition is the dominant source of plasma heating’ is complicated by the temporal nature of an implosion, where the *P*d*V* work on the hot spot that does the heating comes before the time of peak fusion rate, a consideration that is not analogous to MFE. Prior works by Hurricane et al. gave a condition on velocity (*v*_cond_)^2,3^ relative to the plasma conditions, which we slightly modify (Methods) to:

1 $${v}_{{\rm{c}}{\rm{o}}{\rm{n}}{\rm{d}}}(\rho {R}_{{\rm{h}}{\rm{s}}},{T}_{{\rm{i}}})=5.3\times {10}^{25}{\rho R}_{{\rm{h}}{\rm{s}}}\frac{\langle \sigma v\rangle }{{T}_{{\rm{i}}}} > {v}_{{\rm{i}}{\rm{m}}{\rm{p}}}$$

in units of keV, g, cm and s. Here, *ρR*_hs_ is the hot-spot areal density and ⟨*σν*⟩ is the fusion reactivity.

**Fig. 3:**
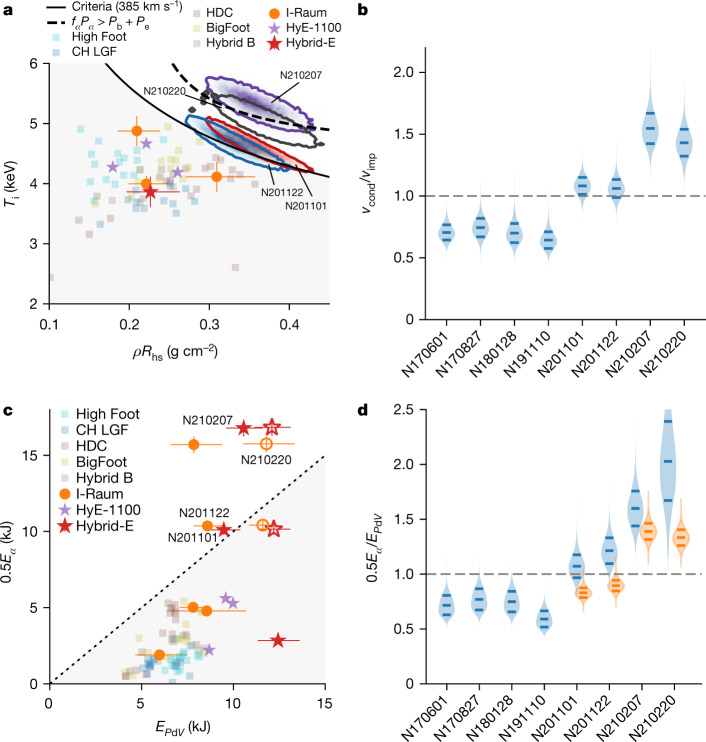
ICF-specific burning-plasma metrics. **a**, Criteria on temperature and hot-spot *ρR* established by Hurricane et al.^3^. Previous experiments are shown as points, and the present four experiments are shown as full probability distributions (red, N201101; blue, N201122; purple, N210207; grey, N210220), with contours enclosing 80% of the distribution. A single contour of equation (1) for *v*_imp_ = 385 km s^−1^ is given by the solid black line. **b**, Probability distribution for experiments exceeding the Hurricane criterion, >1 is a burning plasma. **c**, Criteria on α-heating and *P*d*V* work from a previous work^8^, including estimates from data inferences (solid symbols) and from 2D simulations (open symbols). **d**, Probability distribution for experiments exceeding the Betti criteria. For these experiments distributions are shown for data-inferred *E*_*P*d*V*,hs_ (blue) and using 2D simulations (orange). Error bars in **a**, **c** are 1 s.d. and are shown only for the I-Raum and Hybrid-E points. Historical data are from refs. ^4,14,15,19–21,29–31^.

To evaluate the Hurricane metric, the temperature and areal density of the hot spot, and the implosion velocity, are needed (Methods). The thermonuclear reactivity ⟨*σν*⟩ is a function of the hot-spot conditions, specifically the temperature; we use the ⟨*σν*⟩ evaluation of Bosch and Hale^32^. Figure 3a shows the experiments in hot-spot temperature and areal density parameter space. Previous experiments are shown as points, and the present four experiments are shown as full probability distributions (red, N201101; blue, N201122; purple, N210207; grey, N210220), with contours enclosing 80% of the distribution. In Fig. 3a a single contour of equation (1) for *v*_imp_ = 385 km s^−1^, representative of these experiments, is shown. When evaluating the criteria for the actual inferred velocity of each experiment, with uncertainty, is used. These are the first experiments to exceed the Hurricane criterion, as clearly shown by the probability distributions in Fig. 3b. The likelihood of these four experiments exceeding the criteria is 89% (N201101), 79% (N201122), and 100% for both N210207 and N210220.

Equation (1) should be roughly equivalent to the burning-plasma criteria found by Betti et al. (3.5× yield amplification and 0.5*E*_α_/*E*_*P*d*V*,hs_ > 1)^8^, but for completeness we use both. The first criterion by Betti et al., *Y*_amp_ ≥ 3.5, is satisfied by our inferred yield amplifications given in Extended Data Table 1, inferred with the prescription in ref. ^26^ and from two-dimensional (2D) simulations^6^ using HYDRA^33^. Two quantities are required to evaluate the second Betti et al. burning-plasma metric. The α-deposited energy (*E*_α_) is straightforward as it is simply 20% of the measured total fusion yield (given in Extended Data Table 1), which is approximately 20 kJ for the first two experiments, about 33 kJ for N210207, and about 31 kJ for N210220. The second input for these criteria is the *P*d*V* work done upon the hot spot, which must be inferred; however, such inferences are prone to large uncertainties in the presence of considerable α-heating and bremsstrahlung X-ray losses. We perform this inference in two ways (Methods), first using an analytic hydrodynamic piston model^34^ of an implosion, and second by extracting *P*d*V* work from the 2D radiation–hydrodynamics simulations that best match the experimental observables described in ref ^6^. These two estimates are used to estimate a range in hot-spot *P*d*V* work, and are both plotted in Fig. 3c compared to previous experiments at NIF, with a 1–1 line to denote the burning-plasma regime (above the line). Probability distributions for the metric quantity itself are shown in Fig. 3d. From Betti’s criteria, with the experimental (simulated) *E*_*P*d*V*,hs_, we assess that 74% (0%) and 97% (2%) probability for experiments N201101 and N201122, respectively, are in the burning-plasma regime. The difference in probability reflects the fact that the simulated *P*d*V* work is higher and thus is more pessimistic for satisfying the criteria, with the simulated values for these experiments being below the burning-plasma threshold. With improved performance, experiments N210207 and N210220 are assessed to be in the burning-plasma regime with 100% confidence by both experimental and simulated methodologies. These correspond to an an inferred *Q*_α_ ≈ 1.4–1.6 for experiment N210207, and *Q*_α_ ≈ 1.3–2.0 for experiment N210220.

Several metrics for assessing whether these implosions created a burning-plasma state have been discussed and presented in Extended Data Table 1 and Figs. 2, 3. In each case, burning-plasma likelihoods are calculated by propagating uncertainties in each quantity through the metric (Methods), shown in the figures with likelihood values discussed and summarized in Extended Data Table 1. Quantitatively, we see that the first two (N201101 and N201122) are probably in the burning-plasma regime by all metrics, except Betti’s, when evaluated with the simulated *E*_*P*d*V*,hs_; the most recent experiments (N210207 and N210220) are overwhelmingly likely to have passed this threshold. Qualitatively, our confidence in this conclusion is further increased by the use of multiple independent metrics.

The achievement of a burning-plasma state is key progress towards the larger goal of ‘ignition’ and overall energy gain in inertial fusion. The fusion yields reported here (approximately 0.17 MJ) are lower than the input laser energy (approximately 1.9 MJ), but are nearly equal to the capsule absorbed energy (giving capsule gain of about 0.7–0.8) and are an order of magnitude greater than the input energy transferred to the fusion fuel. Moreover, the total fusion power (5*mP*_α_ where *P*_α_ is the power per unit mass) generated in the two highest performing experiments are at petawatt levels (for example, approximately 1.6 ± 0.2 PW for N210207).

In the burning-plasma regime, self-heating can overtake loss mechanisms, which include bremsstrahlung losses, thermal conductivity and negative *P*d*V* work upon expansion. Simple expressions for the power-balance terms are given in the Methods and values for the four experiments are given in Extended Data Table 1. Here, we use a bremsstrahlung enhancement factor *f*_b_ ≈ 1.15 that is inferred from the data^35^. The first two experiments have self-heating comparable to the radiation losses. An important new regime is when self-heating power (*P*_α_) is greater than both the radiation (*P*_b_) and conduction losses (*P*_e_)—that is, *f*_α_*P*_α_ > *P*_b_ + *P*_e_, where *f*_α_ is the fraction of α-particles stopping in the hot spot^36^. A contour for this regime is shown in Fig. 3a by the black dashed line. Experiment N210220 is close to entering this regime, and we infer that experiment N210207 has entered this regime with 82% likelihood. The level of α-heating in this work is still short of that required for ignition.

**Fig. 4:**
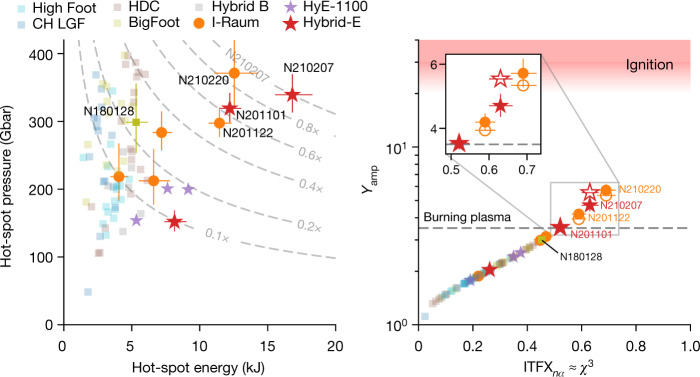
Parameter space relevant for proximity to ignition. Left, hot-spot pressure and energy. The product *P*^2^*E*_hs_ is representative of proximity to ignition; contours of this metric relative to N210207 are shown by the dashed grey curves. Right, yield amplification (*Y*_amp_) versus ITFX_nα_. These are the highest performing ICF experiments so far and the closest to ignition. The inset shows these experiments in detail with both inferred (solid) and simulated (open) *Y*_amp_. Error bars are 1 s.d. and are shown only for the I-Raum and Hybrid-E points, plus shot N180128. Historical data are from refs. ^4,14,15,19–21,29–31^.

To achieve ignition—defined as a yield amplification (*Y*_amp_ ≈ 20–30) consistent with about 1 MJ fusion yield^37^, and then high gain—further progress is needed. Figure 4 shows these experiments in the larger context of ignition, in the parameter space of hot-spot pressure and energy (Fig. 4, left) and in yield amplification versus a Lawson-like parameter called the ‘ignition threshold factor’ experimentally inferred (ITFX)^8,26,38^ for conditions without α-heating (nα) (Fig. 4, right). Figure 4, right, plots this quantity as ${{\rm{ITFX}}}_{{\rm{n}}{\rm{\alpha }}}^{0.34}$, which is approximately equivalent to *χ*_nα_ as defined previously^8^. Proximity to ignition can be gauged qualitatively in terms of the product *P*^2^*E*_hs_ (equivalent to (*ρR*_hs_*T*_i_)^3^), or in terms of ITFX_nα_ or *χ*_nα_ ≈ 1, representing ignition. Figure 4, left, shows contours of *P*^2^*E*_hs_ relative to N210207, showing that this metric has been improved by a factor of several from previous results. From Fig. 4 we clearly see that these four experiments are the closest to ignition, but a further increase in ITFX_*n*α_ from approximately 0.6 → 1 is required. As this Article was being finalized, a new experiment in this series on 8 August 2021 produced approximately 1.35 MJ of fusion yield and capsule gain of approximately 5, breaking all previous records. This was announced by our institution in a press release^39^; this experiment will be described in a future publication.

As discussed in the complementary papers^6,7^, these experiments have clear and specific degradation mechanisms, which can be mitigated for further improvement in performance. More generally, the ICF programme at NIF is pursuing several approaches that can enable additional progress: reducing degradation mechanisms including low-mode asymmetry^40–42^ and radiative losses from mix^35^, further increasing energy coupled to the capsule^4,43^, and improving compression of the fuel^44^.

In conclusion, we have generated in the laboratory a burning- plasma state in which the plasma is predominantly self-heated. This was accomplished using inertial fusion implosions at the US NIF; previous experiments here were just below the threshold for a burning plasma. We increased the capsule scale relative to previous work, increased the coupling efficiency from laser energy to the capsule, and controlled implosion symmetry using new tactics. Four experiments have been conducted that have passed the threshold for a burning plasma by several metrics, with especially high confidence on the most recent two experiments. Additionally, the highest performing experiment (N210207) is in a more stringent regime where the self-heating surpasses energy losses from radiation and conduction. Although these results are short of total energy gain from the system owing to the inherent inefficiencies of ICF, these experiments represent a substantial step towards this goal with record values of parameters that assess our proximity to ignition at NIF. Several promising avenues for further increases in performance are identified and will be pursued by the US inertial fusion programme, in addition to novel physics in the burning-plasma regime such as α-particle-driven processes.

## Methods

### Terminology

Definitions of commonly used mathematical symbols are summarized in Extended Data Table 2.

### Reproducibility

Experiments subsequent to those described in this Article have demonstrated the reproducibility of a burning-plasma state at NIF, with two additional experiments that have performance comparable to the highest-performing experiments in this Article. These newer experiments, N210307 and N210605, were conducted in the Hybrid E platform. N210307 repeated N210207, albeit using a capsule from a different fabrication batch and produced a yield of approximately 145 kJ with an experimentally inferred *Q*_α_ = 1.34 ± 0.07 from the Hurricane criterion. Experiment N210605 reduced the thickness of the ice layer relative to N210207 and resulted in a lower yield (135 kJ) but high ion temperature, with *Q*_α_ = 1.40 ± 0.10, again from the Hurricane criterion. These additional experiments confirm that the burning-plasma state is reproducible at NIF, and full details and analysis on them will be presented in future publications.

### Inferred hot-spot conditions

Hot-spot conditions must be inferred from measured quantities using a model. The simplest hot-spot model is to assume an isobaric volume of uniform conditions, as used in a previous work^14^ between equations 2 and 3, in which case the hot-spot number density is given by

2 $$n=1.2\times {10}^{6}\sqrt{\frac{Y}{\langle \sigma v\rangle {V}_{{\rm{hs}}}\tau }},$$

where *Y* is the fusion yield in J, ⟨*σν*⟩ is the fusion reactivity, which depends on the ion temperature (*T*_i_), *V*_hs_ is the hot-spot volume in cm^3^, and *τ* is the burn duration in s, for equimolar DT mixtures. The remaining hot-spot quantities follow from the inferred density, including the pressure (*P* = (1 + *Z*)*nk*_B_*T*_i_, with *k*_B_ Boltzmann’s constant), hot-spot energy (*E*_hs_ = 1.5*PV*_hs_), and areal density $({\rho R}_{{\rm{h}}{\rm{s}}}=(2.5n/{N}_{{\rm{a}}})\sqrt[3]{3{V}_{{\rm{h}}{\rm{s}}}/4\pi })$.

A more detailed inference is to use a one-dimensional (1D) profile in radius for temperature and density, maintaining the isobaric assumption. A conduction-limited profile follows the expression^45^:

3 $$T(r)={T}_{\min }+({T}_{0}-{T}_{\min }){\left[1-{\left(\frac{r}{{R}_{0}}\right)}^{2}\right]}^{\frac{1}{1+\beta }},$$

where *T*_min_ is the temperature at the boundary, *T*_0_ is the central temperature and *R*_0_ is the hot-spot boundary. *β* is the thermal conductivity power law, 2.5 from classical Spitzer conductivity. Following a previous work^26^ we use a lower value, *β* = 2/3, which accounts for additional physics, dynamical processes and reproduces radiation–hydrodynamics simulations. The density profile is then determined by the isobaric assumption through *P* ∝ *nT* being constant. *T*_min_ is taken as 1 keV leaving *T*_0_, *R*_0_ and *P* as free parameters in the model; the data are compared to synthetic data calculated from this 1D profile with the model parameters adjusted to minimize residuals. As in the zero-dimensional (0D) model, the hot-spot energy simply follows from pressure and volume, and the areal density is the mass density integrated over the inferred radial profile.

In either dimensionality the model’s radius is matched to the experimental measurements, which take a contour of emission level, by calculating synthetic emission images to calculate an equivalent contour radius. The measurements include 2D and three-dimensional (3D) asymmetries, so an equivalent spherical volume, and radius, are calculated using the modal decompositions, where the emission contour measured from the equator (*R*_eq_) and pole (*R*_po_) are

4 $${R}_{{\rm{eq}}}={P}_{0}\{1+\sum _{{\ell }}{\rm{\delta }}{P}_{{\ell }}\times {P}_{{\ell }}(\cos \,\theta )\},$$

5 $${R}_{{\rm{po}}}={M}_{0}\{1+\sum _{m}{\rm{\delta }}{M}_{m}\times \,\cos \,[m(\varphi -{\varphi }_{i})]\},$$

6 $${R}_{{\rm{hs}}}=\frac{{R}_{{\rm{eq}}}{R}_{{\rm{po}}}}{{M}_{0}},$$

where *P*_0_ and *M*_0_ are the average measured sizes from each view, δ*P*_*ℓ*_ (δ*M*_*m*_) is the relative modal amplitude, often referred to as *P*_*ℓ*_/*P*_0_ with the nomenclature above chosen for clarity. *P*_*ℓ*_ are the Legendre polynomials, and *ϕ*_*m*_ are the orientation of the azimuthal modes. *R*_*hs*_ is the hot-spot radius as a function of both *θ* and *ϕ*, which is integrated to obtain the volume. Here, the dominant parameters are *P*_0_, *M*_0_ and δ*P*_2_, which are given in ref. ^7^.

Implosion velocity (*v*_imp_) is inferred using a rocket model of the implosion^46^ constrained by both supporting experiments, especially in-flight radiography, and the measured time of peak nuclear production on each experiment. The inferred yield amplification given in Extended Data Table 1 is a function of the measured yield, shell compression and fuel mass (*m*_fuel_); both the velocity and *Y*_amp_ inferences use the prescription given in a previous work^26^. The fuel kinetic energy then follows from $\frac{1}{2}{m}_{{\rm{fuel}}}{v}_{{\rm{imp}}}^{2}$. Our techniques for inferring the *P*d*V* work done on the fuel are discussed in the following section.

A comparison of inferred values using 0D and 1D models are shown in Extended Data Table 3. Inferred pressures are highly consistent between these calculations, whereas hot-spot energies and areal densities are higher in the 1D model owing to substantial mass near the 1 keV temperature cut-off.

### Inferring *G*_fuel_

The total fusion yield produced by a mass (*m*) of DT, over a characteristic confinement time, *τ*, is *Y* ≈ 5*mP*_α_*τ*—with *P*_α_ = 8.2 × 10^24^*ρ*⟨*σν*⟩ in GJ g^−1^ s^−1^ the specific DT fusion power for a given mass density, *ρ*, of DT with reaction rate ⟨*σν*⟩—and the internal energy in that DT is *E*_hs_ = *c*_DT_*mT*_i_. Therefore, one can write (O.A.H. et al., manuscript in preparation)

7 $${G}_{{\rm{fuel}}}=\frac{Y}{{E}_{P{\rm{d}}V,{\rm{tot}}}}\approx \frac{\frac{Y}{{E}_{{\rm{hs}}}}}{1+\frac{{E}_{{\rm{fuel}}}}{{E}_{{\rm{hs}}}}-\frac{q}{10}\frac{Y}{{E}_{{\rm{hs}}}}},$$

with

8 $$\frac{Y}{{E}_{{\rm{hs}}}}\approx 4.6\times {10}^{26}P\frac{\langle \sigma v\rangle }{{T}^{2}}\tau ,$$

where *P* is in Gbar, *T*_i_ in keV, and *τ* in s. In equation (7), the total energy delivered by *P*d*V* work, *E*_*P*d*V*,tot_, is determined from the hot spot and compressed, but cold, DT fuel energy at stagnation, *E*_hs_ and *E*_fuel_, respectively, at peak compression. The last term in the denominator represents a correction for additional energy retained by self-heating of the fuel from α-particle deposition but not then lost as bremsstrahlung. So, *E*_*P*d*V*,tot_ ≈ *E*_hs_ + *E*_fuel_ − *qY*/10, where *q* is a ‘quality’ factor, 0 ≤ *q* ≤ 1, measuring the ability of the implosion to retain self-heating energy (O.A.H. et al., manuscript in preparation). Here we use *q *≈ 0.7, inferred from simulations, and the factor of 10 results from one-fifth of the fusion energy released as α-particles and half of those produced up until the time of peak fusion burn. Albeit generally arrived at in a different fashion than above, the product *P*(⟨*σν*⟩/*T*^2^)*τ* is Lawson’s^9^ parameter for ignition. Figure 2a uses the useful reaction-rate approximation $\langle \sigma v\rangle \approx 4.2\times {10}^{-20}{T}_{{\rm{i}}}^{3.6}$ (in units of cm^3^ s^−1^ for ion temperature range 3.5 < *T*_i_ < 6.5 keV) to simplify the abscissa.

An expression for the fuel gain is given in equation (7). The yield is measured and the hot-spot energy is inferred as described in the previous section. Precisely determining the cold-fuel energy from data is not straightforward. For the purposes of this analysis we actually require the total *P*d*V* work done on all the DT. This is at a minimum the fuel kinetic energy and internal energy at peak velocity, which are both inferred. This neglects any work done by the inflowing remaining ablator material on the fuel, which can occur in these implosions. In this case the hot-spot energy is more than half the previous estimate; in this scenario we assume equipartition between the hot spot and cold fuel to evaluate equation (7).

### Inferred *P*d*V* work

The primary uncertainty in the Betti metric^8^ is in the inference of *P*d*V* work on the hot spot. Here we use three methodologies: two inferences using an analytic model, and a direct extraction of *P*d*V* work from simulations that match the experimental observables.

We use the hydrodynamic piston model of an implosion described previously^34^. This analytic model abstracts the implosion process using opposed pistons to represent the imploding shell. In spherical geometry, the stagnation pressure from this mechanical work on the hot spot is given by (equation 24in ref. ^34^):

9 $${P}_{{\rm{piston}}}=\frac{\rho {\rm{\delta }}{R}_{{\rm{ave}}}{v}_{{\rm{imp}}}^{2}}{{R}_{{\rm{hs}}}}(1-{f}^{2}\,),$$

where *ρ*δ*R*_ave_ is the average shell areal density, calculated from the measured neutron ‘down-scattered ratio’ (DSR) using the relation *ρ*δ*R*_ave_ ≈ 19.3DSR, *v*_imp_ is the implosion velocity and *R*_hs_ is the average hot-spot radius (which can be obtained from the volume, *V*_hs_, given in Extended Data Table 1). The factor *f* ^2^ represents the effect of mode-1 asymmetry and is a measure of the residual kinetic energy (kinetic energy that is never converted into internal energy) in the implosion.

From the piston pressure we obtain the hot-spot internal energy (*E*_hs_) from

10 $${E}_{{\rm{hs}}}=\frac{3}{2}{P}_{{\rm{piston}}}{V}_{{\rm{hs}}}.$$

In the absence of α-heating (which adds energy to the hot-spot) and radiative X-ray losses, or when α-heating exactly balances X-ray losses, then *E*_hs_ = *E*_*P*d*V*,hs_. For low yield amplification implosions (*Y*_amp_ < 1.5), X-ray losses dominate over α-heating energy gains, so *E*_hs_ < *E*_*P*d*V*,hs_. For higher yield amplification implosions (*Y*_amp_ > 2), α-heating energy gains start to dominate over X-ray losses, so *E*_hs_ > *E*_*P*d*V*,hs_. The estimated values for these four experiments are given in Extended Data Table 4 as the piston methodology.

We can also estimate the stagnated fuel mass in a similar fashion, using

11 $${m}_{{\rm{shell}}}=4{\rm{\pi }}{R}_{{\rm{hs}}}^{2}\,\rho {\rm{\delta }}{R}_{{\rm{ave}}},$$

which allows us to then estimate the total mass that stagnates from *m*_shell_ + *m*_hs_, with *m*_hs_ from the hot-spot inferences described earlier. We then estimate the *P*d*V* work from

12 $${E}_{P{\rm{d}}V,{\rm{hs}}}=0.73{{\rm{KE}}}_{{\rm{fuel}}}\frac{{m}_{{\rm{shell}}}+{m}_{{\rm{hs}}}}{{m}_{{\rm{fuel}}}},$$

where *m*_fuel_ is the initial fuel mass. The factor of 0.73 is derived from 1D simulations in which the imploding mass stagnates efficiently, and we drop the residual kinetic energy factor *f* ^2^ because the inferred shell mass does not include non-stagnated material. This estimate leads to smaller estimates of *E*_*P*d*V*,hs_ than the first empirical estimate, and are given in Extended Data Table 4 as the stagnated mass estimate.

For analysis of previously published campaigns we use the simple relation *E*_*P*d*V*,hs_ ≈ (0.5–0.7)KE_fuel_(1 − *f* ^2^), this is easy to evaluate with the available data and the factor 0.5–0.7 accounts for a wide range of 1D to 2D/3D behaviour observed on past experiments. For comparison, the proportionality constant inferred from the first methodology (equation (10)) is between 0.60 and 0.73 for our four experiments.

We also use radiation–hydrodynamics simulations to estimate the *P*d*V* work done on these implosions. The first simulation-based methodology is to use 2D simulations with degradation mechanisms that match the observed performance, and interrogate the work done upon the mass elements that form the hot spot to infer *E*_*P*d*V*,hs_. The simulation methodology is described in ref. ^6^, and the values of *E*_*P*d*V*,hs_ for this method are given in Extended Data Table 4. The same fusion performance can be generated with varying application of degradation mechanisms that either degrade *E*_*P*d*V*,hs_ or do not; an estimate of the 2D simulation uncertainty of ±0.5 kJ is estimated by studying multiple simulations.

A similar energy-balance analysis can be done with 1D simulations, in which the work done upon the hot spot is well defined with a Lagrangian mesh. The 1D simulations are tuned to match the measured yields, but are expected to underestimate *E*_*P*d*V*,hs_ since they cannot properly incorporate residual kinetic energy. This estimate is given in Extended Data Table 4 as an upper bound.

We have thus develop four methodologies for estimating *E*_*P*d*V*,hs_. In the main analysis we use a combination of the empirical piston model estimate as the more pessimistic data-based inference, and use the 2D simulated *E*_*P*d*V*,hs_ as the most robust computational description of the experiments.

### Modified Hurricane metric

At peak burn, the time rate of change of hot-spot volume, d*V*/d*t*, is nearly zero, and therefore so is the heating rate, so time integration is needed. Mathematically, a statement of a burning plasma appropriate for ICF is

13 $${\int }_{0}^{{t}_{{\rm{pf}}}}{P}_{{\rm{\alpha }}}{\rm{d}}t > -{\int }_{0}^{{t}_{\min V}}\frac{P}{m}{\rm{d}}V,$$

where *t*_pf_ is the time of peak fusion rate, and *t*_min*V*_ is the time of minimum hot-spot volume.

The integrals in equation (13) are easily approximated^2^ without knowing the details of the actual implosion using the mathematical method of steepest descent; assuming that the thermodynamic quantities of interest, such as *T*, *P*, *ρ*, and so on, are impulsive, being highly peaked around the time of stagnation. Ultimately, the solution to equation (13), in terms of only burn-average hot-spot areal density, *ρR*_hs_, *T*_i_ and *v*_imp_ is equation (1) after a correction to the original derivation.

A recent note from our colleagues at Los Alamos^47^ discovered an arithmetic error in the derivation of the criteria as published in ref. ^3^. The error is in going from equation 8 to equation 9 in ref. ^3^, in which the conversion to peak temperature (*T*_0_) to burn-averaged temperature (*T*_hs_) should be, for *n *≈ 4,

14 $$\begin{array}{c}{\frac{\langle \sigma v\rangle }{{T}_{0}}|}_{{T}_{0}}\approx {\left(\frac{n+1}{n}\right)}^{\frac{n-1}{2}}{\frac{\langle \sigma v\rangle }{T}|}_{{T}_{{\rm{hs}}}}\\ \,\approx \,1.40{\frac{\langle \sigma v\rangle }{T}|}_{{T}_{{\rm{hs}}}}\,.\end{array}$$

Additionally, we now believe that the inclusion of the fraction of α-particles stopping in the hot spot (*f*_α_) in ref. ^3^ was inappropriate. When considering the temperature evolution of a defined mass—for example, the self-heating criterion in equation (17)—this is necessary because *f*_α_ is fundamentally the fraction of α-particle energy deposited into that mass. On the other hand, the burning-plasma criteria is one on the energy of the hot spot,

15 $${E}_{{\rm{hs}}}={c}_{{\rm{DT}}}{m}_{{\rm{hs}}}{T}_{{\rm{hs}}},$$

and α-particles that escape the hot spot still contribute to its energy via generation of additional hot-spot mass, as seen by examining the time derivative of the above:

16 $$\begin{array}{ccc}\frac{{\rm{d}}{E}_{{\rm{hs}}}}{{\rm{d}}t} & = & {c}_{{\rm{DT}}}\left({m}_{{\rm{hs}}}\frac{{\rm{d}}{T}_{{\rm{hs}}}}{{\rm{d}}t}+\frac{{\rm{d}}{m}_{{\rm{hs}}}}{{\rm{d}}t}{T}_{{\rm{hs}}}\right)\\  & = & {m}_{{\rm{hs}}}\,{f}_{{\rm{\alpha }}}{Q}_{{\rm{\alpha }}}+{m}_{{\rm{hs}}}(1-{f}_{{\rm{\alpha }}}){Q}_{{\rm{\alpha }}}\\  & = & {m}_{{\rm{hs}}}{Q}_{{\rm{\alpha }}}.\end{array}$$

Therefore, the inclusion of *f*_α_ in a burning-plasma criterion is inappropriate. We note that not including an *f*_α_ factor is consistent with other criteria, for example, ref. ^8^. With these two modifications to the criterion published in ref. ^3^ we use a new criterion (equation (1)). This modified criterion is slightly more restrictive for the burning-plasma threshold in the regime relevant to these experiments.

### Model uncertainties for Hurricane’s metric

The Hurricane metric^3^ depends on more quantities than the Betti metric, although these quantities are more straightforward to infer than *E*_*P*d*V*,hs_. The metric reduces to equation (1) where *ρR*_hs_ and *v*_imp_ are inferred as described previously, and *T*_i_ is measured. ⟨*σν*⟩ contains some systematic uncertainty from the evaluation used. Data uncertainties are well defined for *T*_i_ and in the inference of *ρR*_hs_ and *v*_imp_, and are propagated as described in the next section; the inferred *ρR*_hs_ can also vary between models, which will be discussed.

Equation (1) depends on the fusion reactivity; in this work we use the evaluation from Bosch and Hale^32^. Recent publications have presented alternative evaluations^48^ which differ by about 2%. We note that the inferred $\rho R\propto 1/\sqrt{\langle \sigma v\rangle }$ from equation (2), so the condition in equation (1) depends on the reactivity as $1/\sqrt{\langle \sigma v\rangle }$. *f*_α_ is also weakly increasing with *ρR*, leading to the condition being slightly less than square-root dependent on ⟨*σν*⟩, so this criterion has <1% uncertainty from the choice of ⟨*σν*⟩ evaluation.

The Hurricane criterion is sensitive to the inferred hot-spot *ρR*, which can vary between models depending on the spatial dependence of *ρ*. As shown in Extended Data Table 3, the 0D and 1D hot-spot models agree quite well. We also check these values using a 3D reconstruction of the hot-spot density and temperature profiles (a yet unpublished method of L. Divol, but briefly described in ref. ^35^): for N201101 this gives a value of *ρR*_hs_ ≈ 0.36–0.38 g cm^−2^ to the 1-keV contour for N201101 and *ρR*_hs_ ≈ 0.35–0.36 g cm^−2^ for N201122. These values are consistent with the simple models described earlier.

### Self-heating regime

The hot-spot per unit mass power balance is:

17 $${c}_{{\rm{DT}}}\frac{{\rm{d}}T}{{\rm{d}}t}={f}_{{\rm{\alpha }}}{P}_{{\rm{\alpha }}}-{f}_{{\rm{b}}}{P}_{{\rm{b}}}-{P}_{{\rm{e}}}-\frac{P}{m}\frac{{\rm{d}}V}{{\rm{d}}t},$$

which describes the temporal evolution of the temperature (*T*) in terms of the balance of self heating (*P*_α_) versus bremsstrahlung (*P*_b_) and electron conduction (*P*_e_) losses plus *P*d*V* work. Here electron conduction losses are calculated relative to a hot-spot boundary that is defined relative to a fraction of the peak burn rate or a specified ion temperature. Thermal conduction cools the hot spot while increasing the mass of the hot spot. Because the fusion burn rate is more strongly dependent on the temperature of the spot than its mass in the temperature range achieved by compression alone, α-heating must provide sufficient heating for the hot-spot temperature to increase in the presence of this conduction into an increasing mass. Hot-spot volume change, d*V*/d*t*, is negative on implosion, increasing *T*. During expansion the *P*d*V* term becomes an energy loss term. The bremsstrahlung loss can be enhanced beyond the emission of clean DT by the presence of high-*Z* contamination of the DT (that is, mix), by a fraction *f*_b_. In equation (17), *f*_α_ is the fraction of α-particles stopped in the hot spot, evaluated using fits with modern stopping-power theory^36^.

### Uncertainty analysis

We perform uncertainty analysis for all hot-spot quantities by propagating the normally distributed uncertainties in measured quantities through the 0D and 1D models described earlier. The model input parameters are those that fully describe the system, and are constrained by the measured yield, ion temperature, burn widths (from both X-rays and γ-rays), and volume from the 17% contour of neutron emissivity. Distributions of model parameters are generated using Markov chain Monte Carlo (MCMC), calculated with the tensorflow^49^ probability package. The log-likelihood function for MCMC is defined by the measurements and calculated with the log-likelihood function

18 $$-\frac{1}{2}\sum _{i}{\left(\frac{{m}_{i}-{y}_{i}}{{\rm{\delta }}{y}_{i}}\right)}^{2},$$

which is summed over all observables (*i*) where *m*_*i*_ is the model value, *y*_*i*_ is the measured value and δ*y*_*i*_ is the uncertainty in the measurement. This methodology produces full distributions of the model parameters including any correlations, from the model parameter distributions we generate full distributions of all hot-spot parameters, some of which exhibit correlation, such as in the temperature and areal density required to evaluate the Hurricane metric, which are partially anti-correlated (evident in Fig. 3a). Other inferences, such as the implosion velocity or kinetic energy, are treated with normally distributed uncertainties that are uncorrelated with the hot-spot inferences.

### Power-balance relations

In evaluating the power-balance relations relevant to equation (17) we use the following expressions for the individual terms:

19 $${P}_{{\rm{\alpha }}}=8.2\times {10}^{24}\rho \langle \sigma v\rangle ,$$

20 $${P}_{{\rm{b}}}=3.1\times {10}^{7}\rho \sqrt{T},$$

21 $${P}_{{\rm{e}}}=5.9\times {10}^{3}\frac{{T}^{3.5}}{\rho {R}^{2}}.$$

In these expressions the specific powers are given in units of GJ g^−1^ s^−1^ and thus are multiplied by the inferred hot-spot mass to obtain power. *ρ* is the hot-spot mass in g cm^−3^, ⟨*σν*⟩ is the fusion reactivity evaluated as a function of temperature in cm^3^ s^−1^, *T* is the temperature in keV, and *ρR* is the hot-spot areal density in g cm^−2^. The self-heating power *P*_α_ is multiplied by the fraction of α-particle energy deposited in the hot spot (*f*_α_) using the evaluation published in ref. ^36^; for all four experiments, *f*_α_ ≈ 0.77–0.80.

## Online content

Any methods, additional references, Nature Research reporting summaries, source data, extended data, supplementary information, acknowledgements, peer review information; details of author contributions and competing interests; and statements of data and code availability are available at 10.1038/s41586-021-04281-w.

## Figures and Tables

**Extended Data Table 1:**
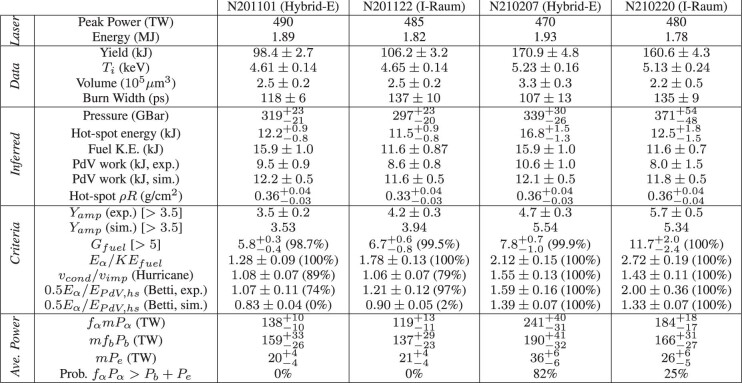
Scalar metrics for these experiments

Data, inferred metrics and burning-plasma criteria for these four experiments. Percentages indicate probabilities. The criteria >1 corresponds to a burning plasma, except for *Y*_amp_ and *G*_fuel_, with the threshold for those criteria given in brackets. Errors are formal ±1*σ* standard deviations.

**Extended Data Table 2:**
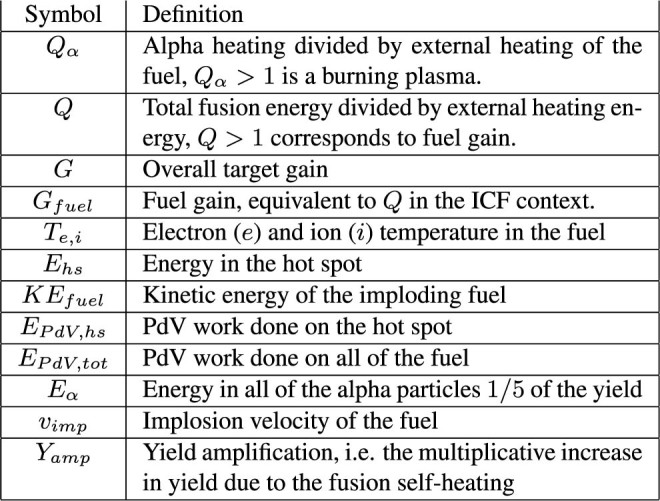
Symbols

Definitions for symbols used in this paper.

**Extended Data Table 3:**
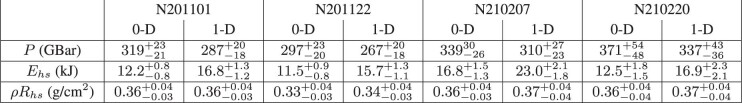
Hot-spot models

Comparison of inferred quantities from 0D and 1D hot-spot models.

**Extended Data Table 4:**
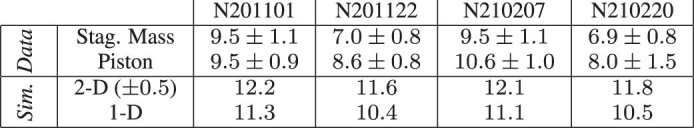
*P*d*V* work methodologies

Inferred hot-spot *P*d*V* work (in kJ) by different methodologies, ordered from smallest to largest estimates.

## Data Availability

Raw data were generated at the National Ignition Facility. Derived data supporting the findings of this study are available from the corresponding authors upon request.
