# Publisher Correction: Burning plasma achieved in inertial fusion

**DOI:** 10.1038/s41586-022-04607-2

**Published:** 2022-03-16

**Authors:** A. B. Zylstra, O. A. Hurricane, D. A. Callahan, A. L. Kritcher, J. E. Ralph, H. F. Robey, J. S. Ross, C. V. Young, K. L. Baker, D. T. Casey, T. Döppner, L. Divol, M. Hohenberger, S. Le Pape, A. Pak, P. K. Patel, R. Tommasini, S. J. Ali, P. A. Amendt, L. J. Atherton, B. Bachmann, D. Bailey, L. R. Benedetti, L. Berzak Hopkins, R. Betti, S. D. Bhandarkar, J. Biener, R. M. Bionta, N. W. Birge, E. J. Bond, D. K. Bradley, T. Braun, T. M. Briggs, M. W. Bruhn, P. M. Celliers, B. Chang, T. Chapman, H. Chen, C. Choate, A. R. Christopherson, D. S. Clark, J. W. Crippen, E. L. Dewald, T. R. Dittrich, M. J. Edwards, W. A. Farmer, J. E. Field, D. Fittinghoff, J. Frenje, J. Gaffney, M. Gatu Johnson, S. H. Glenzer, G. P. Grim, S. Haan, K. D. Hahn, G. N. Hall, B. A. Hammel, J. Harte, E. Hartouni, J. E. Heebner, V. J. Hernandez, H. Herrmann, M. C. Herrmann, D. E. Hinkel, D. D. Ho, J. P. Holder, W. W. Hsing, H. Huang, K. D. Humbird, N. Izumi, L. C. Jarrott, J. Jeet, O. Jones, G. D. Kerbel, S. M. Kerr, S. F. Khan, J. Kilkenny, Y. Kim, H. Geppert Kleinrath, V. Geppert Kleinrath, C. Kong, J. M. Koning, J. J. Kroll, M. K. G. Kruse, B. Kustowski, O. L. Landen, S. Langer, D. Larson, N. C. Lemos, J. D. Lindl, T. Ma, M. J. MacDonald, B. J. MacGowan, A. J. Mackinnon, S. A. MacLaren, A. G. MacPhee, M. M. Marinak, D. A. Mariscal, E. V. Marley, L. Masse, K. Meaney, N. B. Meezan, P. A. Michel, M. Millot, J. L. Milovich, J. D. Moody, A. S. Moore, J. W. Morton, T. Murphy, K. Newman, J.-M. G. Di Nicola, A. Nikroo, R. Nora, M. V. Patel, L. J. Pelz, J. L. Peterson, Y. Ping, B. B. Pollock, M. Ratledge, N. G. Rice, H. Rinderknecht, M. Rosen, M. S. Rubery, J. D. Salmonson, J. Sater, S. Schiaffino, D. J. Schlossberg, M. B. Schneider, C. R. Schroeder, H. A. Scott, S. M. Sepke, K. Sequoia, M. W. Sherlock, S. Shin, V. A. Smalyuk, B. K. Spears, P. T. Springer, M. Stadermann, S. Stoupin, D. J. Strozzi, L. J. Suter, C. A. Thomas, R. P. J. Town, E. R. Tubman, C. Trosseille, P. L. Volegov, C. R. Weber, K. Widmann, C. Wild, C. H. Wilde, B. M. Van Wonterghem, D. T. Woods, B. N. Woodworth, M. Yamaguchi, S. T. Yang, G. B. Zimmerman

**Affiliations:** 1 Lawrence Livermore National Laboratory, Livermore, CA USA; 2 Los Alamos National Laboratory, Los Alamos, NM USA; 3 Laboratoire pour l’utilisation des Lasers Intenses chez École Polytechnique, Palaiseau, France; 4 Laboratory for Laser Energetics, University of Rochester, Rochester, NY USA; 5 General Atomics, San Diego, CA USA; 6 Massachusetts Institute of Technology, Cambridge, MA USA; 7 SLAC National Accelerator Laboratory, Menlo Park, CA USA; 8 Atomic Weapons Establishment, Aldermaston, UK; 9 Diamond Materials, Freiburg, Germany

**Keywords:** Nuclear fusion and fission, Laser-produced plasmas

Correction to: *Nature* 10.1038/s41586-021-04281-wPublished online 26 January 2022

In the version of this article initially published, an author’s name was omitted from the list. C. Trosseille (Lawrence Livermore National Laboratory, Livermore, CA, USA) was missing from the author list, and their contribution to the X-ray framing camera was also not shown. The amendments have been made to the html and PDF versions of the article

